# An mHealth-Delivered Sexual Harm Reduction Tool (PartyPack) for Men Who Have Sex With Men in Malaysia: Usability Study

**DOI:** 10.2196/48113

**Published:** 2023-08-24

**Authors:** Kamal Gautam, Kiran Paudel, Jerome Jacobs, Jeffrey A Wickersham, Wan Mohd Ikhtiaruddin, Iskandar Azwa, Rumana Saifi, Sin How Lim, Roman Shrestha

**Affiliations:** 1 Department of Allied Health Sciences University of Connecticut Storrs, CT United States; 2 AIDS Program Yale School of Medicine New Haven, CT United States; 3 Centre of Excellence for Research in AIDS Faculty of Medicine University of Malaya Kuala Lumpur Malaysia; 4 Infectious Diseases Unit, Department of Medicine Faculty of Medicine University of Malaya Kuala Lumpur Malaysia

**Keywords:** chemsex, party and play, sexualized drug use, PartyPack, harm reduction tool, men who have sex with men, Malaysia, health promotion, sexual health, mHealth intervention, HIV prevention

## Abstract

**Background:**

Chemsex—the use of psychoactive drugs to enhance the sexual experience—is an increasing phenomenon globally. Despite the increasing burden and associated harms of chemsex, evidence-based interventions (ie, behavioral and pharmacological) for chemsex users are nonexistent.

**Objective:**

In this study, we assessed the usability and acceptability of a mobile health (mHealth)–delivered safer chemsex package (“PartyPack”) as a sexual harm reduction strategy among men who have sex with men in Malaysia—a setting where chemsex is becoming increasingly prevalent.

**Methods:**

This study is part of a larger smartphone app-based intervention (ie, JomPrEP; University of Connecticut) designed to improve access to HIV prevention services among Malaysian men who have sex with men. A total of 50 participants were recruited from the Greater Kuala Lumpur region of Malaysia to use the JomPrEP app, which included a feature allowing participants to order PartyPack, for 30 days (March-April 2022). The usability and acceptability of the PartyPack were assessed using self-report, app analytics, and exit interviews (n=20).

**Results:**

Overall, 8% (4/50) of participants reported having engaged in chemsex in the past 6 months; however, engagement in condomless sex (34/50, 68%) and group sex (9/50, 18%) was much higher. A total of 43 (86%) participants ordered PartyPack, of which 27 (63%) made multiple orders during the 30 days. Most participants (41/43, 95%) reported being satisfied with the PartyPack order feature in the app, with 91% (39/43) indicating the order and tracking process was easy. Thematic data exploration further revealed important information for understanding (eg, items included in the package, use of mHealth platform to order package, and discreetness of the PartyPack box and order and delivery) and refining the logistical preferences (eg, using branded items and allowing customization during order).

**Conclusions:**

Our findings provide strong evidence of the usability and acceptability of a mHealth-delivered safer chemsex package as a potential sexual harm reduction tool among this underserved population. Replication in a study with a larger sample size to test the efficacy of the PartyPack is warranted.

## Introduction

Chemsex, “the use of psychoactive drugs before or during planned sexual activity to sustain, enhance, disinhibit, or facilitate the experience,” is an increasing phenomenon globally [1,2]. This form of sexualized drug use is common among men who have sex with men (MSM) and has been linked to the use of specific drugs such as methamphetamine, mephedrone, gamma hydroxybutyrate, and gamma-butyrolactone [2,3]. The prevalence of chemsex among MSM ranges from 3% to 41% across countries [4-10]. Empirical studies have found that MSM who engage in chemsex exhibit high-risk sexual behaviors, such as condomless anal sex, group sex, and having multiple sexual partners [3,6,11,12]. Furthermore, there is strong evidence linking chemsex to severe health consequences, such as withdrawal symptoms, overdose, heart failure, depression, anxiety, and various sexually transmitted infections (STIs), including HIV and bloodborne infections such as hepatitis C [13-17].

Similar to the Global North, chemsex is steeply rising across Asia, including Malaysia [11,18-20]. Several reasons influencing the decision of MSM to practice chemsex include the direct benefit of using the drug to enhance sexual pleasure, persuasion from peers, and external prejudice toward MSM [21-23]. Unfortunately, Malaysia’s strict drug laws and criminalizing same-sex sexual behavior make it challenging for MSM to seek health care services [22,24]. Moreover, no behavioral or pharmacotherapeutic interventions are placed to address this problem. Notably, reports indicate that those who engage in chemsex often seek information about harm reduction services in a digital platform or within their community rather than consulting health care providers [25].

In response to this unmet need, we designed the PartyPack, a safer chemsex package as a sexual harm reduction tool for MSM in Malaysia. It includes various harm reduction supplies to equip individuals with relevant information and tools to reduce risks associated with chemsex. These supplies include condoms and lube for safer sex, antiseptic for safer fisting, oral rehydration salts to prevent dehydration, and various hygiene and fun products to create safer conditions for chemsex. As part of our implementation effort, we incorporated a feature within our existing app, JomPrEP, allowing users to order PartyPack [26]. The JomPrEP app primarily focuses on HIV prevention among MSM in Malaysia and includes features like HIV self-test kit orders, e-consultations, and pre-exposure prophylaxis (PrEP) medication orders.

In this study, we conducted a mixed methods study among MSM to assess the usability and acceptability of a mobile health (mHealth)–delivered PartyPack as a sexual harm reduction strategy among MSM in Malaysia.

## Methods

### Study Design and Participants

This study is part of a larger smartphone app-based intervention (ie, JomPrEP) designed to improve access to HIV prevention services among Malaysian MSM [26]. A total of 50 participants were recruited from the Greater Kuala Lumpur region of Malaysia to use the JomPrEP app, which included a feature allowing participants to order PartyPack, for 30 days (March-April 2022). We partnered with the Centre of Excellence for Research in AIDS at the University of Malaya, Kuala Lumpur, Malaysia, to conduct this study.

The eligibility criteria included (1) being 18 years or older; (2) identifying as a cisgender man; (3) self-reporting an HIV-negative or HIV status unknown at screening; (4) not having used PrEP previously for HIV prevention; (5) owning a smartphone; and (6) currently residing in the Greater Kuala Lumpur region.

### Study Procedures

The participants were recruited using both in-person and digital strategies. Flyers were distributed and posted at local partner organizations, and social media platforms, including MSM-focused Facebook pages and the geosocial networking app (ie, Hornet) were used for recruitment. Interested individuals were directed to the study website for a brief description and web-based screening.

After meeting the enrollment criteria, eligible participants were asked to provide electronic informed consent and undergo baseline assessment. Study staff then assisted enrolled participants with downloading the JomPrEP app and provided them with brief instructions on the purpose of the app, including an overview of how to use the app to order and track the safer chemsex package (ie, PartyPack). Participants were requested to use the app for 30 days and to complete a posttest survey at the end of the study period. To restrict access to JomPrEP to the study participants, participants were provided with a single-use registration code needed to gain access to the app.

**Figure 1:**
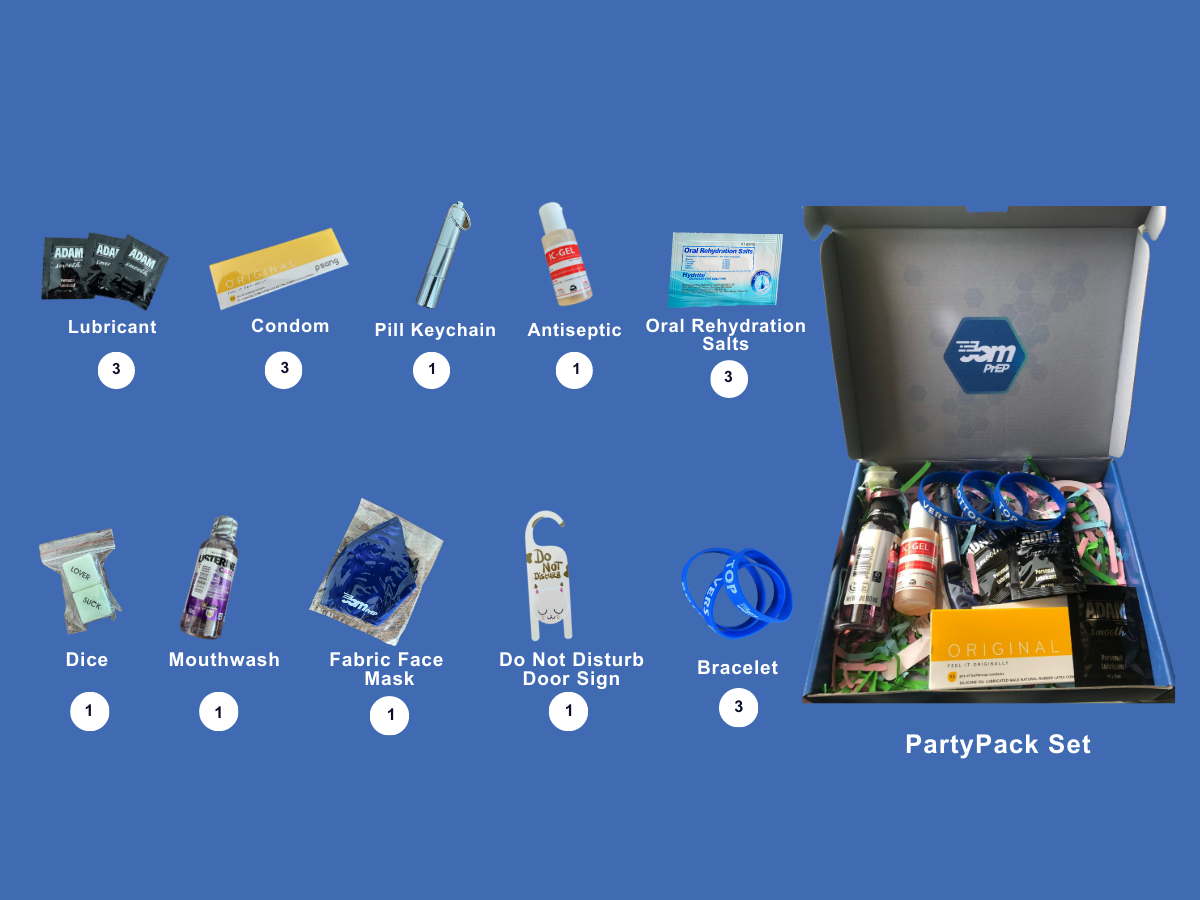
Items included in the PartyPack and their quantity.

Upon downloading the app, participants were asked to complete an onboarding process, which included creating log-in credentials. They were then redirected to the JomPrEP landing screen (ie, home screen), which contains several icons representing key app functions, including an “Order” feature that allows users to order and track the delivery of PartyPack. The PartyPack contains various items along with their corresponding quantities, as shown in Figure 1. Participants could simply provide the mailing address for contactless PartyPack delivery at their preferred location within 2 business days and be able to track the shipment on the app, all free of cost.

### Ethics Approval

This study was approved by the institutional review board at the University of Connecticut (H22-0049) with an institutional reliance agreement with the University of Malaya.

### Study Measures

The survey assessment was conducted digitally and self-administered using Qualtrics. The survey collected participants’ demographic characteristics, including age, ethnicity, educational status, relationship status, income, depressive symptoms, substance use, sexual history, HIV or STI-testing practices, and past use of PrEP and post-exposure prophylaxis (PEP).

Satisfaction with the PartyPack feature was measured with a single-item question: “How satisfied are you with the ordering PartyPack feature of the app?” Participants responded using a 5-point Likert scale (from 1=not at all satisfied to 5=extremely satisfied). The rating of satisfaction was coded as “satisfied” if responded as “very satisfied” and “extremely satisfied” (excluded options: “not at all satisfied,” “slightly satisfied,” or “moderately satisfied”). Similarly, the usability of the PartyPack feature was measured with a single-item question: “How easy or difficult was it to order a PartyPack from the app?” Participants responded using a 4-point Likert scale (1=very difficult to 4=very easy). The participants’ rating of the level of difficulty was coded as “easy” if responded as “very easy” (excluded options: “very difficult,” “difficult,” and “neutral”).

Additionally, we conducted one-to-one exit interviews using videoconferencing technology with 20 (40%) participants to obtain feedback on PartyPack features, especially their usefulness, preferences for specific items, and feedback for further refinement.

### Statistical Analysis

All quantitative data were managed and analyzed using SPSS Statistics (version 28; IBM Corp). Means for continuous variables and frequencies for categorical variables were calculated to describe the participants. The usability and acceptability of the PartyPack were based on descriptive statistics from the app analytics and acceptability measure. For qualitative data, all the exit interviews were audio-recorded, transcribed, and analyzed. The comments and issues were grouped and categorized according to common themes relative to specific app functions by 2 coders (and agreed upon by all authors). Dedoose (version 9.0.54; SocioCultural Research Consultants, LLC) was used throughout to assist in data management and analysis.

## Results

### Participant Characteristics

The participant characteristics are described in Table 1. The mean age of the participants was 27.9 (SD 5.3) years. The majority were single (36/50, 72%), Malay (26/50, 52%), university graduates (34/50, 68%), or living with other people in a house or apartment (36/50, 72%). Nearly all participants (49/50, 98%) reported being tested for HIV, with 78% (39/50) being tested within 6 months. More than half (26/50, 52%) of the participants reported using HIV self-testing, whereas only 10% (5/50) reported using PrEP. Regarding sexual behavior in the past 6 months, 94% (47/50) of participants reported engaging in anal sex with another man, while only 32% (16/50) reported consistent condom use. Additionally, 8% (4/50) reported engaging in chemsex, and 18% (9/50) reported engaging in group sex.

**Table 1:** Characteristics of participants (N=50).

Variables		Frequency
Age (years), mean (SD)		27.9 (5.3)
**Ethnicity, n (%)**		
	Malaya	26 (52)
	Others	24 (48)
**University graduate^a^, n (%)**		
	No	16 (32)
	Yes	34 (68)
**Relationship status, n (%)**		
	Single	36 (72)
	Partner	14 (28)
Monthly income, mean (SD)		RM 3553.40 (2985.90) [US $837.97 (704.14)]
**Living status, n (%)**		
	Alone	14 (28)
	Living with others	36 (72)
**Tested for HIV (past 6 months), n (%)**		
	No	11 (22)
	Yes	39 (78)
**Ever used an HIV self-testing kit, n (%)**		
	No	24 (48)
	Yes	26 (52)
**Previously diagnosed with STI^b^, n (%)**		
	No	27 (54)
	Yes	23 (46)
**Ever used PrEP^c^, n (%)**		
	No	45 (90)
	Yes	5 (10)
**Ever used PEP^d^, n (%)**		
	No	46 (92)
	Yes	4 (8)
**Perceived HIV risk, n (%)**		
	None	6 (12)
	Low	25 (50)
	Moderate	15 (30)
	High	4 (8)
**Ever injected drugs, n (%)**		
	No	49 (98)
	Yes	1 (2)
**Engaged in anal sex (past 6 months), n (%)**		
	No	3 (6)
	Yes	47 (94)
**HIV serodiscordant relationship (past 6 months), n (%)**		
	No	47 (94)
	Yes	3 (6)
**Consistent condom use (past 6 months), n (%)**		
	No	34 (68)
	Yes	16 (32)
**Engaged in group sex (past 6 months), n (%)**		
	No	41 (82)
	Yes	9 (18)
**Engaged in chemsex^e^ (past 6 months), n (%)**		
	No	46 (92)
	Yes	4 (8)

^a^Includes college, university, and professional degree.

^b^STI: sexually transmitted infection (eg, gonorrhea, chlamydia, and syphilis).

^c^PrEP: pre-exposure prophylaxis.

^d^PEP: postexposure prophylaxis.

^e^Use of psychoactive substances before or during sexual activity.

### Uptake and Evaluation of the PartyPack

Table 2 displays information on PartyPack orders and app user satisfaction with the ordering procedure. Of the participants, 43 (86%) ordered PartyPack, with 27 (63%) making multiple orders of the PartyPack. Of these 43 users, 41 (95%) were satisfied with the ordering process, and 39 (91%) found ordering PartyPack easy via the JomPrEP app.

**Table 2:** Participants’ uptake, satisfaction, and level of difficulty in ordering PartyPack via the app.

Variables		Participants (N=50), n (%)
**Users ordering PartyPack**		43 (86)
	Users making repeat PartyPack orders	27 (63)
	Total PartyPack orders	83
	Satisfied with the “Ordering PartyPack” feature^a^	41 (95)
	Ease of ordering PartyPack^b^	39 (91)

^a^Very satisfied and extremely satisfied (not included: not at all satisfied, slightly satisfied, and moderately satisfied).

^b^Very easy (not included: very difficult, difficult, and neutral).

### Exit Interviews

#### Overview

In the exit interviews, 20 participants participated in in-depth interviews. Of them, 19 had ordered PartyPack at least once, and 1 participant had never ordered PartyPack. Four overarching themes emerged from the thematic analysis: (1) reasons for ordering and not ordering the PartyPack; (2) usability and acceptability of the PartyPack; (3) feedback and suggestions for improvements; and (4) considerations for future use.

#### Theme 1: Reasons for Ordering and Not Ordering the PartyPack

During the exit interviews, we discovered that the name “PartyPack” had a persuasive impact on the participants. Additionally, including useful items in the package further motivated them to place and use orders.

I often go to parties, and the name PartyPack and the useful items included really reflects cuteness as well as kinkiness. That's why I ordered it without any hesitation.

Participant 5

Some participants mentioned that they ordered PartyPack because it had a variety of items and was provided for free. They were convinced to try it and see what it had to offer.

I order the PartyPack because it has some useful items for me, and the best part is, it's free.

Participant 16

One participant said they did not order PartyPack because they did not know this feature existed in the app. But, after it was explained, they became curious about PartyPack.

I went through the order feature but only noticed HIV Self-Test kit order. Therefore, I ordered only the test kit. I didn’t even know the PartyPack feature existed… So, what was inside of the PartyPack.

Participant 11

#### Theme 2: Usability and Acceptability of the PartyPack

Most participants consistently expressed positive experiences regarding the app's ordering process. They described it as “very easy” with just a simple click, appreciating the inclusion of clear instructions.

I think the order is very, very easy to make... it's just a click, and also inside the app, it's showing a lot of the instructions.

Participant 13

Several PartyPack recipients used or tried the PartyPack items. Products received positively were condoms, lubricants, pill keychains, and antiseptics. They were widely used and highly accepted.

I used the condom and the lubricant, of course. And we get the antiseptic... very useful.

Participant 15

The most useful items for me are the condom, lubricant, and the keychain for pills.

Participant 1

Participants had mixed reactions to including items like masks, dice, and rubber bracelets. Some participants were pleased to have these fun and basic products available, while others either chose not to use them or disagreed with their inclusion depending on their level of sexual activity and personal preferences.

While it's nice to have a customized mask, I personally don't wear cloth masks.

Participant 3

You can just get rid of that rubber bracelet, as well as the dice, but the mask I would say is very useful.

Participant 8

The mask and the mouthwash are most useful currently and rest are not useful because I’m not sexually active currently.

Participant 19

The most useful I would say the condoms and the lube, but what I like the most is the sassy and fun dice.

Participant 9

The app platform was regarded as a valuable tool for maintaining privacy and discretion when accessing PartyPack. Participants highlighted the significance of receiving these items privately through the app, particularly for individuals who faced challenges or discomfort in obtaining them through traditional channels. The app’s ability to provide a discreet means of acquiring safer sex items was a key factor contributing to its acceptability.

Receiving free sexual health items through the mail delivery in a discreet packet is really valuable for me… it helps people like me who have some sort of reservation buying items, especially like lubricants publicly.

Participant 4

Accessing sexual health items is something I do pretty easily anyway. But for someone who doesn't, I think receiving those kinds of things privately via the mobile app would be really useful.

Participant 1

#### Theme 3: Feedback and Suggestions for Improvements

Many participants suggested adding a feature that allows users to customize their PartyPack items, thus giving them more control over what they receive.

It would be nice to have the option to choose what to include in the PartyPack.

Participant 6

A few participants suggested that including a well-known brand in PartyPack would make it more appealing. They believed that including a well-known brand would enhance the overall value and attractiveness of PartyPack.

The PartyPack would be more useful and attractive if it included a well-known brand.

Participant 9

#### Theme 4: Considerations for Future Use

Many participants emphasized that their interest and decision to order PartyPack in the future when the app is publicly available would be influenced by its cost.

For me, I think, it [future order] depends on the price, because now it's free testing, but it might be charged in the future. So, I think it's much more depending on price.

Participant 16

## Discussion

### Principal Results

To our knowledge, this is the first study to explore the use of a safer chemsex package to reduce the sexual risks of engagement in chemsex among MSM. Our study provides empirical evidence to support the feasibility and acceptability of a mHealth-delivered safer chemsex package for MSM in Malaysia, as chemsex users already use smartphones for geosocial networking and searching harm reduction services [1,4,12,23,25], making mHealth interventions a practical and effective way to intervene. The findings also offer the use of mHealth platforms, such as smartphone apps, to support and provide harm reduction commodities for MSM participants to reduce the negative impacts of their engagement in chemsex and promote safe sexual behaviors.

A high level of ease in using the mHealth platform, satisfaction, and repeated PartyPack orders indicate that the service is well received by the target population. The participants particularly appreciated the mHealth platform and discreet delivery process, as MSM in Malaysia faces stigma and discrimination due to the criminalization of same-sex sexual activity and drug use. Accessing safer chemsex packages and consultation services in traditional health care settings is contested because of the fear of stigma, discrimination, and legal repercussions associated with same-sex behavior and chemsex practices [27-29]. However, mHealth tools can offer confidential and less stigmatizing access to these resources, making it easier to overcome the barriers that prevent access to traditional clinic-based care for marginalized populations, such as MSM. Participants are also likely to uptake PartyPack features if they are made available and affordable in the future.

### Comparison With Prior Work

Our findings are consistent with prior research as they reaffirm the usability and acceptability of harm-reduction interventions in promoting sexual health among MSM. Mimiaga et al [30] found that a harm reduction intervention that provided nonintrusive prevention and education activities such as condoms, lubricants, and coupons for free HIV and STI testing were acceptable among the majority (80%) of the MSM participants who attended sex parties. Similarly, APCOM's TestBKK distributes condoms, lubricants, and HIV awareness materials through social media platforms. The distribution is discreet to ensure privacy and nondiscrimination, and the demand is high among MSM who practice chemsex [31,32]. However, our study emphasizes the use of the mobile app to distribute harm-reduction materials. This is a relatively new approach that has not been fully explored in prior research.

Although there is a consensus regarding the usability and acceptability of PartyPack, some participants in the exit interview provided valuable recommendations for refinement, for example, adding an option to customize the items in the pack and using well-known brands. In addition, some participants suggested removing masks from PartyPack because of the JomPrEP logo. Considering these suggestions in future iterations of the PartyPack feature is essential for enhancing its acceptability and effectiveness. In addition to PartyPack, it is crucial to add other harm-reduction features to the app to provide holistic support to prevent drug-related harm and mitigate the exacerbation of HIV, other STIs, and mental health symptoms among MSM engaged in chemsex. These features may include e-consultations for drug-related harm, the option to order HIV self-testing kits and PrEP, and mental health and psychosocial support. As it may be challenging for current chemsex users to stop drug use within a short period, educating and empowering them to practice safer sex, even in drug-influenced situations, can be a practical strategy to reduce associated risks.

### Limitations

This study has several limitations such as a small sample size, short-term follow-up, and a single-arm design, making it neither powered nor designed to evaluate efficacy. The participants were subject to selection bias and were recruited through Facebook or a dating app. In addition, not all participants had engaged in chemsex, and the study was limited to the Greater Kuala Lumpur area, potentially limiting the generalizability of the findings. There is also a chance of social desirability bias, and the free PartyPack service may have overestimated uptake.

### Conclusions

The use of safer packages, such as PartyPack, is a feasible and acceptable harm-reduction measure for MSM in Malaysia. Using the mHealth platform has added significant value to interventions. Its discreet delivery to the doorsteps of users ensures that marginalized populations feel safe and less vulnerable to legal or social consequences. Promising outcomes have been reported, highlighting the need for large-scale studies to test the efficacy of PartyPack in real-world settings.

## Abbreviations

mHealth: mobile health
MSM: men who have sex with men
PEP: post-exposure prophylaxis
PrEP: pre-exposure prophylaxis
STI: sexually transmitted infection

## Data Availability

The data sets generated and analyzed during this study are available from the corresponding author upon reasonable request.

## References

[1] Stuart D (2016). A chemsex crucible: the context and the controversy. J Fam Plann Reprod Health Care.

[2] McCall H, Adams N, Mason D, Willis J (2015). What is chemsex and why does it matter?. BMJ.

[3] Tomkins A, George R, Kliner M (2019). Sexualised drug taking among men who have sex with men: a systematic review. Perspect Public Health.

[4] Maxwell S, Shahmanesh M, Gafos M (2019). Chemsex behaviours among men who have sex with men: a systematic review of the literature. Int J Drug Policy.

[5] Kohli M, Hickson F, Free C, Reid D, Weatherburn P (2019). Cross-sectional analysis of chemsex drug use and gonorrhoea diagnosis among men who have sex with men in the UK. Sex Health.

[6] Drückler Susanne, van Rooijen MS, de Vries HJC (2018). Chemsex among men who have sex with men: a sexualized drug use survey among clients of the sexually transmitted infection outpatient clinic and users of a gay dating app in Amsterdam, the Netherlands. Sex Transm Dis.

[7] Sewell J, Cambiano V, Speakman A, Lampe FC, Phillips A, Stuart D, Gilson R, Asboe D, Nwokolo N, Clarke A, Rodger AJ (2019). Changes in chemsex and sexual behaviour over time, among a cohort of MSM in London and Brighton: findings from the AURAH2 study. Int J Drug Policy.

[8] Logan L, Fakoya I, Howarth A, Murphy G, Johnson AM, Rodger AJ, Burns F, Nardone A (2019). Combination prevention and HIV: a cross-sectional community survey of gay and bisexual men in London, October to December 2016. Euro Surveill.

[9] Frankis J, Flowers P, McDaid L, Bourne A (2018). Low levels of chemsex among men who have sex with men, but high levels of risk among men who engage in chemsex: analysis of a cross-sectional online survey across four countries. Sex Health.

[10] Ruiz-Robledillo N, Ferrer-Cascales R, Portilla-Tamarit I, Alcocer-Bruno C, Clement-Carbonell V, Portilla J (2021). Chemsex practices and health-related quality of life in Spanish men with HIV who have sex with men. J Clin Med.

[11] Bourne A, Reid D, Hickson F, Torres-Rueda S, Weatherburn P (2015). Illicit drug use in sexual settings ('chemsex') and HIV/STI transmission risk behaviour among gay men in South London: findings from a qualitative study. Sex Transm Infect.

[12] Shrestha R, Lim SH, Altice FL, Copenhaver M, Wickersham JA, Saifi R, Ab Halim MA, Naning H, Kamarulzaman A (2020). Use of smartphone to seek sexual health information online among Malaysian men who have sex with men (MSM): implications for mHealth intervention to increase HIV testing and reduce HIV risks. J Community Health.

[13] Bourne A, Ong J, Pakianathan M (2018). Sharing solutions for a reasoned and evidence-based response: chemsex/party and play among gay and bisexual men. Sex Health.

[14] Daskalopoulou M, Rodger A, Phillips AN, Sherr L, Speakman A, Collins S, Elford J, Johnson MA, Gilson R, Fisher M, Wilkins E, Anderson J, McDonnell J, Edwards S, Perry N, O'Connell R, Lascar M, Jones M, Johnson AM, Hart G, Miners A, Geretti A, Burman WJ, Lampe FC (2014). Recreational drug use, polydrug use, and sexual behaviour in HIV-diagnosed men who have sex with men in the UK: results from the cross-sectional ASTRA study. Lancet HIV.

[15] Giorgetti R, Tagliabracci A, Schifano F, Zaami S, Marinelli E, Busardò FP (2017). When "Chems" meet sex: a rising phenomenon called "ChemSex". Curr Neuropharmacol.

[16] Pufall EL, Kall M, Shahmanesh M, Nardone A, Gilson R, Delpech V, Ward H, Positive Voices study group (2018). Sexualized drug use ('chemsex') and high-risk sexual behaviours in HIV-positive men who have sex with men. HIV Med.

[17] Íncera-Fernández D, Gámez-Guadix M, Moreno-Guillén S (2021). Mental health symptoms associated with sexualized drug use (Chemsex) among men who have sex with men: a systematic review. Int J Environ Res Public Health.

[18] Maviglia F, Wickersham JA, Azwa I, Copenhaver N, Kennedy O, Kern M, Khati A, Lim SH, Gautam K, Shrestha R (2022). Engagement in chemsex among men who have sex with men (MSM) in Malaysia: prevalence and associated factors from an Online National Survey. Int J Environ Res Public Health.

[19] Lim SH, Mburu G, Bourne A, Pang J, Wickersham JA, Wei CKT, Yee IA, Wang B, Cassolato M, Azwa I (2017). Willingness to use pre-exposure prophylaxis for HIV prevention among men who have sex with men in Malaysia: findings from an online survey. PLoS One.

[20] Paudel K, Gupta S, Gautam K, Wickersham JA, Khati A, Azwa I, Ha T, Shrestha R (2023). High interest in long-acting injectable pre-exposure prophylaxis (LAI-PrEP) for HIV prevention among men who have sex with men (MSM): result from a nationwide survey in Malaysia. J Community Health.

[21] Wang H, Jonas KJ, Guadamuz TE (2023). Chemsex and chemsex associated substance use among men who have sex with men in Asia: a systematic review and meta-analysis. Drug Alcohol Depend.

[22] Lim SH, Akbar M, Wickersham JA, Kamarulzaman A, Altice FL (2018). The management of methamphetamine use in sexual settings among men who have sex with men in Malaysia. Int J Drug Policy.

[23] Newland J, Kelly-Hanku A (2021). A qualitative scoping review of sexualised drug use (including Chemsex) of men who have sex with men and transgender women in Asia. APCOM.

[24] (2021). Briefing note: chemsex and harm reduction for gay men and other men who have sex with men. Harm Reduction International.

[25] Dennermalm N, Scarlett J, Thomsen S, Persson KI, Alvesson HM (2021). Sex, drugs and techno - a qualitative study on finding the balance between risk, safety and pleasure among men who have sex with men engaging in recreational and sexualised drug use. BMC Public Health.

[26] Shrestha R, Altice FL, Khati A, Azwa I, Gautam K, Gupta S, Sullivan PS, Ni Z, Kamarulzaman A, Phiphatkunarnon P, Wickersham JA (2023). Clinic-integrated smartphone app (JomPrEP) to improve uptake of HIV testing and pre-exposure prophylaxis among men who have sex with men in Malaysia: mixed methods evaluation of usability and acceptability. JMIR Mhealth Uhealth.

[27] Earnshaw VA, Jin H, Wickersham J, Kamarulzaman A, John J, Altice FL (2014). Exploring intentions to discriminate against patients living with HIV/AIDS among future healthcare providers in Malaysia. Trop Med Int Health.

[28] Earnshaw VA, Jin H, Wickersham JA, Kamarulzaman A, John J, Lim SH, Altice FL (2016). Stigma toward men who have sex with men among future healthcare providers in Malaysia: would more interpersonal contact reduce prejudice?. AIDS Behav.

[29] Jin H, Earnshaw VA, Wickersham JA, Kamarulzaman A, Desai MM, John J, Altice FL (2014). An assessment of health-care students' attitudes toward patients with or at high risk for HIV: implications for education and cultural competency. AIDS Care.

[30] Mimiaga MJ, Reisner SL, Bland S, Cranston K, Isenberg D, Driscoll MA, VanDerwarker R, Mayer KH (2010). “It's a quick way to get what you want”: a formative exploration of HIV risk among urban Massachusetts men who have sex with men who attend sex parties. AIDS Patient Care STDS.

[31] Nevendorff L, Puspoarum T, ThanhTung D, Kaplan K (2021). Chemsex in Asia: a community manual on sexualised substance use among MSM. APCOM.

[32] (2021). Demand for HIV prevention during Covid-19: testBKK experience. APCOM.

